# Availability and advertising of electronic cigarettes in two Russian cities following implementation of comprehensive tobacco advertising restrictions

**DOI:** 10.18332/tid/115794

**Published:** 2020-01-20

**Authors:** Lisa P. Lagasse, Ashley S. Grant, Marela Kay R. Minosa, Ryan D. Kennedy, Joanna E. Cohen

**Affiliations:** 1 Institute for Global Tobacco Control, Johns Hopkins Bloomberg School of Public Health, Baltimore, United States

**Keywords:** electronic cigarettes, tobacco control, advertising and promotion, surveillance, Russia

## Abstract

**INTRODUCTION:**

Electronic cigarettes (e-cigarettes) continue to gain popularity globally. Jurisdictions with comprehensive tobacco control policies, which limit the use and availability of combustible tobacco products but do not regulate e-cigarettes (as in Russia), may be vulnerable to the expansion of the e-cigarette market.

**METHODS:**

Using McNemar’s test conducted in STATA, this observational study assessed changes between 2014 and 2016 in the availability of e-cigarettes across 239 retail outlets in Moscow and St Petersburg. Also, this study characterized the presence of retail advertising and promotion of e-cigarettes in 2016.

**RESULTS:**

Between 2014 and 2016, the availability and display of e-cigarettes increased within traditional tobacco product retail venues (27.6% in 2014 vs 51.9% in 2016; p<0.01).

**CONCLUSIONS:**

Observations indicate that there has been an increase in the proportion of retailers selling and displaying e-cigarettes.

## INTRODUCTION

Tobacco use in the Russian Federation (Russia) is a critical public health concern. According to World Health Organization estimates, the prevalence of adult smokers in Russia is among the highest in the European region: 49.8% of men and 14.5% of women over the age of 15 years were current tobacco smokers in 2016^1^. Tobacco is the third leading cause of premature death in Russia, following high blood pressure and high cholesterol^2^. In response to the heavy burden of tobacco-related morbidity and mortality, Russia has strengthened its tobacco control policy in recent years^3^. In February 2013, the Federation Council of the Russian Federation approved Federal Law No.15-FZ^4^, a comprehensive tobacco control law. The law was implemented in two phases. Beginning in June 2013, the law restricted smoking in public spaces and banned all forms of tobacco advertising, promotion, and sponsorship. The following year, beginning in June 2014, the display of tobacco products in retail outlets was prohibited and the sale of tobacco was restricted to outlets that include a shopping area for customers. Prior to 2014, tobacco sales were permitted in grocery and convenience stores, gas stations, and kiosks. Following implementation of the federal tobacco control law, kiosks were excluded from tobacco retailing.

Importantly, electronic nicotine delivery systems (ENDS), or e-cigarettes, are not covered under Federal Law No. 15-FZ, and thus restrictions on the display and marketing of conventional tobacco products do not extend to e-cigarettes, leaving a potentially critical policy gap. Russia is an important global market for e-cigarettes. About twelve per cent (11.9%) of adults^5^ and more than a quarter (28.6%) of adolescents^6,7^ in Russia have tried e-cigarettes.

Russia’s high prevalence of tobacco use and strong tobacco control law may push both smokers and vendors to seek alternative products in order to cope with recent restrictions on sales, use, and marketing of conventional tobacco products – providing an opportunity for the expansion of the e-cigarette market. Indeed, research demonstrates that exposure to e-cigarette advertising is associated with greater use of these products^8,9^. This study examines changes in e-cigarette availability prior to and following full implementation of a comprehensive tobacco control policy in Russia and describes the prevalence of advertising and promotion of e-cigarettes, comparing across retailer types.

## METHODS

Trained researchers conducted observations in retail venues in the two most populous Russian cities: Moscow and St Petersburg. These cities were selected based on guidance from in-country partners, who pointed to the importance of these jurisdictions in driving policy making for the larger Federation. The same retail venues (chain supermarkets, independent markets/convenience stores/gas stations, classified as ‘independent stores’, and kiosks) were visited at two time periods: first in April to May 2014 – immediately prior to full implementation of the national tobacco control law – and again in March to April 2016. These retail venues were initially identified using a walking protocol, details are given elsewhere^10^.

At both time periods, researchers used mobile phones, equipped with a proprietary customizable mobile data collection software app, to complete a checklist capturing the product availability (i.e. products available for sale at the retail venue) along with the presence and types of displays (i.e. visibility through the window, in the cashier zone, or other location) for both conventional tobacco products and e-cigarettes. Data were also collected to observe the advertising and promotion (e.g. signage, discounts, gifts with purchase) of conventional tobacco products. In 2016, items detailing the advertising and promotion of e-cigarettes were added to the observations. These data were not collected for e-cigarettes in 2014. Details of the data collection instrument and software are given elsewhere^10^.

### Data analysis

The availability and display of e-cigarettes for each observational time period, as well as the advertising and promotional elements observed among retailers selling e-cigarettes in 2016, were characterized using descriptive statistics. McNemar’s test assessed the significance of changes in the availability and display between retailer types; analysis was conducted using STATA 14.0^11^. Store-level data were excluded from the analysis when observations were missing for the 2014 time period (n=7) (i.e. data collector was asked to leave the store before observation was complete) or the store had ceased to operate by the 2016 data collection period (n=53). No new tobacco retailers were added to the sample. Data were analysed in aggregate across both cities.

## RESULTS

In 2014, researchers visited 295 retail venues; during the 2016 data collection period, researchers determined that 51 retail venues (34 in Moscow and 17 in St Petersburg) had ceased to operate and 5 were missing observations, leaving a sample of 239 retailer venues (116 locations in Moscow and 123 locations in St Petersburg). In total, the 2016 sample included 96 supermarkets, 82 independent stores, and 61 kiosks. The proportion of retail venues selling e-cigarettes increased between 2014 and 2016 (27.6% in 2014 vs 51.9% in 2016; p<0.01), with the greatest change observed in supermarkets (14.6% vs 55.2%; p<0.01), followed by independent stores (31.7% vs 47.6%; p=0.01) (Table 1). While there was an increase in availability of e-cigarettes among kiosks (42.4% vs 52.5%), the change was not found to be significant (p=0.18).

**Table 1:** E-cigarette availability and display, by store type and observation year (N=239)

*Store type*	*Availability*			*Display*			*Advertising & Promotion*
	*2014*	*2016*	*p*	*2014*	*2016*	*p*	*2016*
All stores	**66 (27.6)**	**124 (51.9)**	**<0.01**	**47 (19.7)**	**98 (41.0)**	**<0.01**	73 (30.5)
Supermarkets (n=96)	**14 (14.6)**	**53 (55.2)**	**<0.01**	**10 (10.4)**	**48 (50.0)**	**<0.01**	41 (42.7)
Independent stores (n=82)	**26 (31.7)**	**39 (47.6)**	**0.01**	19 (23.2)	28 (34.1)	0.06	8 (9.8)
Kiosks (n=61)	26 (42.6)	32 (52.5)	0.18	18 (29.5)	22 (36.1)	0.34	24 (39.3)

Values represent number and percentage, n (%). Bolded p-values meet statistical significance of p≤0.05. Independent stores include convenience stores and gas stations.

Similarly, e-cigarette product displays increased in both cities across all retail venues between 2014 and 2016 (19.7% vs 41.0% overall; p<0.01), but the only significant increase was observed in supermarkets (10.4% vs 50.0%; p<0.01). Increased product displays were observed for e-cigarettes sold in independent stores (23.2% vs 34.1%) and kiosks (29.5% vs 36.1%), but the differences were not significant (p>0.05). Nearly a third of retailers (30.5%) had some form of e-cigarette advertising and promotion; almost all advertising was signage (29.1%), such as posters, banners, or shelf liners that represented a specific brand.

A preliminary analysis identified a greater increase in the availability of e-cigarettes between 2014 and 2016 among the retailers observed in Moscow compared to St Petersburg (36.2% vs 7.3%; p<0.001). Similarly, the presence of e-cigarette advertising was also significantly greater in Moscow in 2016 (45.7% vs 16.3%; p<0.001).

## DISCUSSION

In an effort to address the country’s high burden of tobacco use, Russia has made important changes in its tobacco control policy. The national law made substantial progress in the protection of public health by prohibiting smoking in public places and banning the marketing and display of conventional tobacco products. However, findings from this study indicate that e-cigarettes may represent an important policy gap, as these products are not covered by the national tobacco control law.

We found that availability and advertising of these products increased in the two years following passage of the law. Observations demonstrate that many of the retailers who sold e-cigarettes highlighted these products through display; advertising and promotion of e-cigarettes was present in the form of posters, banners, and shelf liners. Previous research demonstrates that e-cigarette advertisements at the point-of-sale can increase cigarette cravings among daily smokers and reduce intentions to remain abstinent among former smokers^12,13^. For this reason, the exclusion of e-cigarettes from the national tobacco control law may have significant negative consequences undermining the intended aims of the legislation.

Findings from this study highlight the ways in which e-cigarettes were promoted following passage of Federal Law 15-FZ. However, it should be noted that the data from 2014 did not include marketing of e-cigarettes, and were limited to product availability and display. In addition, this study did not assess in depth the differences between Moscow and St Petersburg; future studies may examine city-level differences in the implementation and impact of comprehensive tobacco control laws. Moreover, due to the observational nature of the data, it is unclear why proportions of independent stores and kiosks did not increase significantly between the data collection periods. Studies evaluating these differences across or between retail vendor types could be the subject of future work.

## CONCLUSIONS

Electronic cigarettes, which are not covered under the Russian comprehensive tobacco control law, may represent a policy gap. Because e-cigarettes are not currently regulated in Russia, it is possible that marketing practices may become more aggressive in the future. This is particularly important for jurisdictions with strong tobacco control policies, as in Russia, where aggressive e-cigarette marketing practices may impede efforts to protect public health. Future research should continue to monitor the prevalence and content of e-cigarette marketing to examine if and how these products are positioned in the marketplace relative to cigarettes and other tobacco products.
